# Treatment Experience of 210 Pediatric Patients With Extraordinary Daytime Urinary Frequency: A Prospective Study

**DOI:** 10.3389/fped.2021.713810

**Published:** 2021-10-28

**Authors:** Yan Li, Ying Zhang, Chao Liu, Xiang Li, Qi Zhou, Chao Sun, Lei Zhang

**Affiliations:** ^1^Pediatric Center, Qilu Hospital of Shandong University, Qingdao, China; ^2^Department of Breast Surgery, Linyi People's Hospital, Linyi, China; ^3^Department of Pediatric Surgery, Qilu Hospital of Shandong University, Qingdao, China

**Keywords:** pediatric extraordinary daytime urinary frequency, pediatric patients, pollakiuria, bladder and bowel dysfunction, treatment, laxative

## Abstract

**Background:** Lactulose can be used to manage chronic constipation and children who are withholding their bowel movements, but no studies are available regarding lactulose to treat pediatric extraordinary daytime urinary frequency (PEDUF). To explore the benefits of different therapeutic regimens (non-drug treatment vs. oral lactulose) in patients with PEDUF.

**Methods:** This prospective study included PEDUF patients admitted to the Pediatric Center of Qilu Hospital of Shandong University (Qingdao) from January 2015 to December 2019. The patients randomized received non-drug treatment (counseling), drug treatment (lactulose), or combination therapy. A therapeutic effect was defined by a decrease of>10% of the urination frequency.

**Results:** A total of 210 patients were included. They were 5.9 ± 0.4 years. There were 98 boys and 112 girls. Among the 210 patients, 82.4% (173/210) of their family members reported symptoms of constipation. Among the three groups, the response rate was 61.4% (43/70) in the non-drug treatment group, 90.0% (63/70) in the drug treatment group, and 91.4% (64/70) in the combination therapy group (*P* < 0.0001).

**Conclusion:** The frequency of constipation in children with PEDUF is high. The use of a laxative, like lactulose, might achieve a high therapeutic response rate in children with PEDUF, higher than counseling alone. That might represent a valuable therapeutic strategy for PEDUF.

## Introduction

Clinically, pediatric extraordinary daytime urinary frequency (PEDUF) is one of the most common symptoms for children with bladder and bowel dysfunction (BBD) (1), which frequently occurs in preschoolers. Healthy children can change from a normal urinary pattern to a pattern characterized by frequent urination, with a frequency of once per 5 min to once per hour and a small volume of urine each time (1). Typically, there are no accompanying signs or symptoms (pain, burning, incontinence, changes in the urinary stream, changes in nocturnal voiding pattern, excessive fluid intake, abnormal urine analysis, or positive urine culture), but most patients have dry stools, difficult defecation, and stool retention (2–4). Generally, the urinary symptoms disappear after falling asleep, and the results of the routine urine test and urinary system ultrasonography are unremarkable (3). Other conditions have to be excluded before diagnosing PEDUF: polydipsia, diabetes, daytime polyuria, urinary tract infection, nephrogenic diabetes insipidus, and viral syndromes (1).

The symptoms of most children can be spontaneously relieved, but sometimes symptoms may recur or persist, seriously interfering with their daily life and leading to psychological disorders, causing great mental burdens on their parents. Currently, the main clinical strategies include defecation management, biofeedback, electrophysiological stimulation, psychological counseling, and medication. Of note, PEDUF has been observed in children with obsessive-compulsive disorder, tic disorders, and Tourette syndrome (5, 6), suggesting that the possibility of such disorders can be considered in the management of PEDUF.

Lactulose is a non-absorbable sugar often used to treat chronic constipation in patients of all ages (7–9), hepatic encephalopathy, and any other diseases that require emptying the intestine. The lactulose dose can be adjusted to achieve the desired effect in various constipation severity levels (7–9). It can also be used for children who develop a fear of their bowel movements and are withholders (7–9). At present, no studies are available regarding lactulose for the treatment of PEDUF.

Therefore, this study aimed to explore the benefits of different therapeutic regimens (non-drug treatment vs. oral lactulose) in patients with PEDUF. The results might help to provide new clinical treatment options for PEDUF.

## Materials and Methods

### Study Design and Patients

This prospective study included PEDUF patients admitted to the Pediatric Center of Qilu Hospital of Shandong University (Qingdao) from January 2015 to December 2019. The diagnostic criteria were (10) (1) a significant increase in the urinary frequency at daytime or in an awaking state, exceeding eight times, (2) distracting attention could alleviate the symptom, (3) the symptoms disappear after falling asleep, (4) no dysuria, fever, polydipsia, and other symptoms, and (5) the results of the routine urine tests and urinary system ultrasound are unremarkable. Children with cognitive disabilities were excluded. This study was approved by the Institutional Review Board of Qilu Hospital, and written informed consent was obtained from each subject before enrollment.

### Data Collection

Questionnaires were issued routinely for PEDUF patients at the first visit (Appendix 1 in Supplementary Material). Systemic examinations were performed to check the external genitalia, the presence or absence of vulvitis, external genital swelling and trauma, etc. for female patients, and balanoposthitis, phimosis, etc. for male patients. The auxiliary examinations included routine urine test (to exclude urinary system infection, hypercalciuria, and other metabolic factors), urinary system ultrasound, residual urine test, kidney, ureter, and bladder X-ray (KUB), rectal diameter (if >3 cm: more intestinal contents and stool retention may be present), urinary system infections, and malformations.

The diagnosis of constipation referred to the Rome III diagnostic criteria (11): (1) two or fewer defecations per week, (2) one or more fecal incontinence per week, (3) history of excessive stool retention, (4) history of painful or hard bowel movements, (5) history of large-diameter stools (rectal diameter ≥ 3 cm in the non-defecating state, and (6) presence of stool clogging to the toilet. Constipation was diagnosed if the patient met at least two items. The Bristol stool scale was used to classify the stool into seven categories: (1) nut-shaped, (2) dry and hard, (3) lumpy, (4) banana-shaped, (5) soft, (6) mushy, and (7) watery. The time of each bowel movement and the classification of stool properties were recorded routinely.

### Therapeutic Regimens

Consenting children who fulfilled the inclusion criteria were randomized to one of the three strategies. (1) Non-drug treatment group: treatment mainly involved relieving the tension and anxiety of the children and their parents, providing corresponding psychological counseling, guiding urination training, prolonging the time of urine holding appropriately or distracting attention, enhancing confidence, and giving appropriate incentives. (2) Drug treatment group: there was no detailed psychological guidance plan, but lactulose was used to increase bowel movements and relieve constipation. Lactulose was given according to body weight. The treatment lasted 5–14 days, based on the investigators' experience. (3) Combination therapy group: the children received lactulose and counseling. Other treatments options were not allowed during the study and would lead to immediate study termination for such participants. For children not reporting constipation, long-term adherence was recommended by improving diet, especially diet structure and eating temperature.

### Main Outcome Measures

All children were followed up in the outpatient clinic. At the first visit, unified health and treatment education was provided, including detailed therapeutic regimens and a voiding diary (Appendix 2 in Supplementary Material). The routine follow-up period was 4 weeks. The main follow-up contents were the changes in the urinary frequency and the time period. The evaluation criteria of the efficacy were (1) cured (daytime urinary frequency was reduced by 70% compared with before treatment or less than eight times), (2) markedly improved (a reduction of 50–70%), (3) slightly improved (a reduction of 10–50%), and (4) ineffective (a reduction of less than 10%). The response rate referred to the percentage of cured and markedly improved cases in the total number of cases.

### Statistical Analysis

Descriptive statistics were used. The rates of patients achieving different response levels were analyzed using the chi-square test in SPSS21.0. *P*-values < 0.05 were considered statistically significant.

## Results

### Characteristics of the Patients

**Figure 1:**
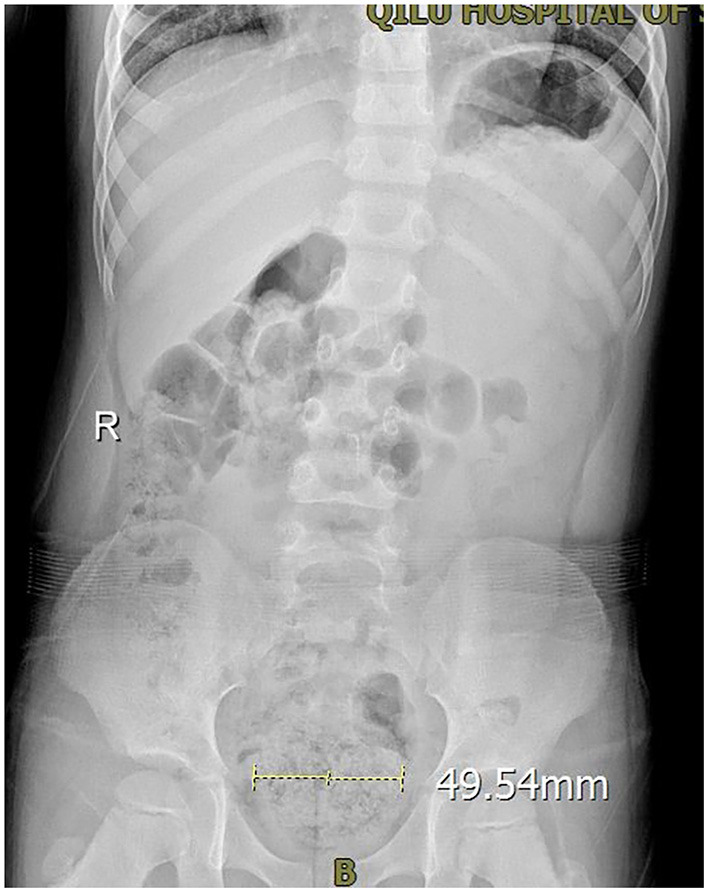
The KUB of a PEDUF patient in an involuntary state showing that the rectum was dilated, and there were many intestinal contents.

There were 210 children in this study, including 98 boys and 112 girls, with a mean age of 5.9 ± 0.4 years (Table 1). All children underwent urinary system ultrasound and routine urine tests to exclude urinary system infections and urinary system malformations. External genital examinations showed no vulvitis and balanoposthitis. Among the 210 children, 10.9% (23/210) had mental stress, and the main reason was the beginning of school or attending kindergarten, 6.2% (13/210) had a history of drinking acidic beverages such as orange juice before frequent urination, 82.4% (173/210) of their family members reported symptoms of constipation (but none was on ongoing medication for constipation), and 14.3% (30/210) of their family members reported one defecation per day and no constipation, but the KUB revealed many intestinal contents, and stool retention or incomplete defecation (Figure 1). Only 3.4% (7/210) had no symptoms of stool retention or incomplete defecation. No children used forbidden medication.

**Table 1:** Clinical characteristics of the 210 patients with PEDUF.

**Characters**	**ALL *N* = 210**
Age, years, mean ± SD	5.9 ± 0.4
Sex, *n* (%)	
Male	98 (46.7)
Female	112 (53.3)
Self-reported constipation, *n* (%)	173 (82.4)
Mental stress, *n* (%)	23 (10.9)
Preferring beverages, *n* (%)	13 (6.2)
KUB, *n* (%)	30 (14.3)

*KUB, kidney, ureter, and bladder X-ray*.

### Treatment Response

**Figure 2:**
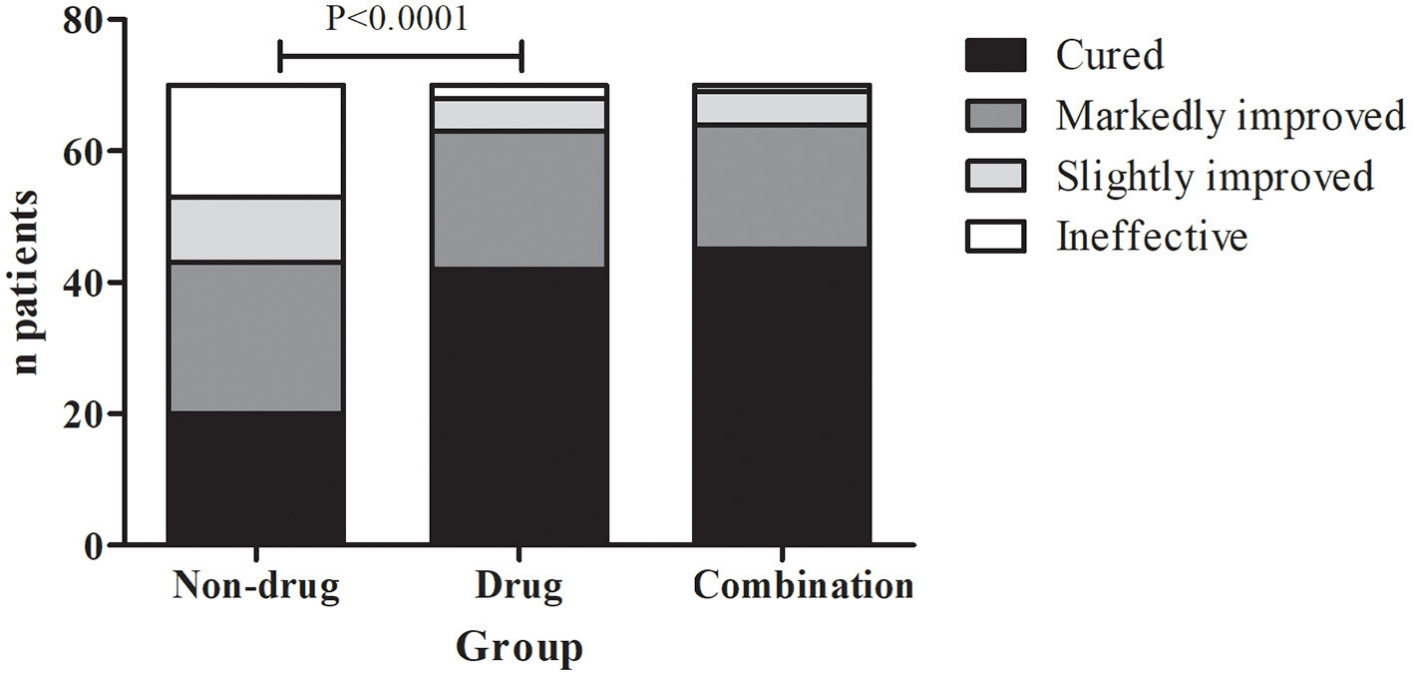
Distribution of the treatment responses in the three groups.

In the non-drug treatment group, 20 were cured, 23 were markedly improved, 10 were slightly improved, and 17 were ineffective, for a response rate of 61.4% (43/70). Those numbers were 42, 21, 5, and 2 in the drug treatment group for a response rate of 90% (63/70). In the combination therapy group, those numbers were 45, 19, 5, and 1, for a response rate of 91.4% (64/70) (Figure 2 and Table 2). The differences among the three groups were significant (*P* < 0.01). The differences between the non-drug treatment and Drug treatment groups were significant (*P* < 0.01).

**Table 2:** Comparison of efficacy.

**Therapeutic regimen**	**Non-drug treatment group**	**Drug treatment group**	**Combined treatment**
Effective	43	63	64
Ineffective	27	7	6
Response rate	61.4%	90%	91.4%

*P < 0.01 among the three groups.*

*Cured: daytime urinary frequency was reduced by 70% compared with before treatment or less than eight times; markedly improved: a reduction of 50–70%; slightly improved: a reduction of 10–50%; ineffective: a reduction of less than 10%. The response rate referred to the percentage of cured and markedly improved cases in the total number of cases*.

## Discussion

PEDUF is the most common symptom of BBD in children, along with constipation (1) and a common disorder in pediatric urology clinics, and was reported by Stephens et al. (3). It frequently occurs in preschoolers and mainly manifests as daytime urinary frequency >1 time/h. In severe cases, patients can urinate once every 5–10 min, with a small urine volume each time, and the symptoms disappear after falling asleep. Most patients have dry stools, difficult defecation, and stool retention (2–4). Lactulose can be used to manage chronic constipation and children who are withholding their bowel movements, but no studies are available regarding lactulose to treat PEDUF. Therefore, this study aimed to explore the benefits of different therapeutic regimens (non-drug treatment vs. oral lactulose) in patients with PEDUF. The results strongly suggest that using a laxative, like lactulose, might achieve a high therapeutic response rate in children with PEDUF, higher than counseling alone. That might represent a valuable therapeutic strategy for PEDUF.

It has been suggested that constipation is closely associated with BBD because the anorectum is closely correlated with lower urinary tract function (1). Therefore, constipation is often associated with bladder dysfunction (12, 13). It has been reported that among children with bladder dysfunction, the incidence of constipation is ~30–88% (14–16). In this study, the incidence of constipation was 82.4%, which was consistent with the literature. The association between bowel dysfunction and bladder dysfunction is called BBD (1). The underlying pathological mechanism of this combined dysfunction remains unclear. Still, it might include the following aspects (17–22): (1) rectal dilation compresses the posterior wall of the urinary bladder, (2) nerve afference of the urethral and anal sphincter has the same functional unit, and (3) stool retention can lead to continuous contraction of the external anal sphincter, which causes improper contraction of the pelvic floor muscles, and then results in the no relaxation of the urethral sphincter and detrusor-urethral sphincter incoordination.

Treatment options for daytime urinary frequency include non-drug treatment, mental and psychological guidance, drug treatment, bladder perfusion, and other methods (12, 13, 20). Non-drug treatments, including psychological counseling, relieve their mental and psychological pressure as much as possible, alleviating tension and anxiety (23, 24). Urination training can allow children to learn to control urination when they have the intention to urinate and prolong the storage time as much as possible to make the bladder normally dilate, starting from delaying urination for 1–2 min, gradually extending to >30 min, and correct the urination posture. At the same time, the parents are encouraged to pay more attention to and accompany their children, distract their attention when symptoms of frequent urination occur, and give appropriate encouragement and rewards when symptoms of frequent urination improve. Still, for young children, compliance with psychological counseling and urination training might remain poor.

Drug treatment mainly uses cholinergic receptor blockers to inhibit the detrusor's overactivity, but it is often accompanied by side effects such as constipation, polydipsia, and blurred vision (2). Bladder perfusion needs a urinary catheter insertion, which is an invasive procedure, so it is difficult for children and their families to accept this method (25). This study found that 82.4% of PEDUF patients had constipation symptoms, and another 14.3% had many intestinal contents on KUB. Hence, oral laxatives can be used to treat constipation, change stool properties, correct poor bowel habits, and significantly improve or cure clinical symptoms in 90% of children. Furthermore, the children had good compliance and no obvious side effects, which deserve clinical application.

In this study, concomitant treatments were now allowed. Therefore, the effects of different combination strategies are unknown. PEDUF treatments should be at a minimal cost while achieving the best effect. Since enema is not well-accepted by children, lactulose might be a good choice since it is an oral treatment and can achieve a good therapeutic effect. Future studies should examine multimodal treatment strategies.

This study has limitations. There was no comparator group using cholinergic receptor blockers because this method was seldom used in children at this hospital. No bladder structural and functional metrics were analyzed because they are not routinely assessed in all children. Although this was a prospective study, the sample size was not calculated. Future studies should address those limitations. In addition, no follow-up was performed. PEDUF is an intermittent situation. Most of the symptoms should significantly reduce or disappear with age. Future studies could look at the effect of repeated treatment to determine the long-term effects of the treatment.

In conclusion, PEDUF appears to be closely associated with constipation. Most previous treatments emphasized psychotherapy and urination training, but young age might be a barrier to compliance and effectiveness. The use of oral laxatives alone, such as lactulose, might achieve good compliance and better therapeutic effects for the management of PEDUF.

## Data Availability Statement

The original contributions presented in the study are included in the article/Supplementary Material, further inquiries can be directed to the corresponding author/s.

## Ethics Statement

The studies involving human participants were reviewed and approved by the Institutional Review Board of Qilu Hospital. Written informed consent to participate in this study was provided by the participants' legal guardian/next of kin.

## Author Contributions

YL conceived and coordinated the study, designed, performed and analyzed the experiments, and wrote the paper. YZ, CL, XL, QZ, CS, and LZ carried out the data collection, data analysis, and revised the paper. All authors reviewed the results and approved the final version of the manuscript.

## Conflict of Interest

The authors declare that the research was conducted in the absence of any commercial or financial relationships that could be construed as a potential conflict of interest.

## Publisher's Note

All claims expressed in this article are solely those of the authors and do not necessarily represent those of their affiliated organizations, or those of the publisher, the editors and the reviewers. Any product that may be evaluated in this article, or claim that may be made by its manufacturer, is not guaranteed or endorsed by the publisher.
