# Supplementary material for: Treatment Experience of 210 Pediatric Patients With Extraordinary Daytime Urinary Frequency: A Prospective Study
Source: Front Pediatr. 2021 Oct 28;9:713810. doi: 10.3389/fped.2021.713810 (PMC8582598; doi:10.3389/fped.2021.713810)
Supplement: Supplementary file 1 [file Data_Sheet_1.DOCX]

**Appendix 1.** Questionnaire of PEDUF patients in the outpatient clinic

**Questionnaire of daytime urinary frequency in the first visit**

**Name:** **Gender** □Male □Female **Age:** **Outpatient No.:**

| Frequency of urination (/day) | □8-16 times □Over 16 times |
| --- | --- |
| Duration of symptoms | □Within 1 week □ 1-2 weeks □ 2 weeks or more |
| Onset time | □Morning □ Afternoon □ Night or before sleep |
| Presence or absence of urgency and dysuria | □Yes □ No |
| Constipation symptoms (multiple choices available) | □No □ 2 or fewer defecation per week;  □1 or more fecal incontinence per week;  □ History of excessive stool retention;  □ History of painful or hard bowel movements; □ Presence of stool clogging to the toilet; |
| 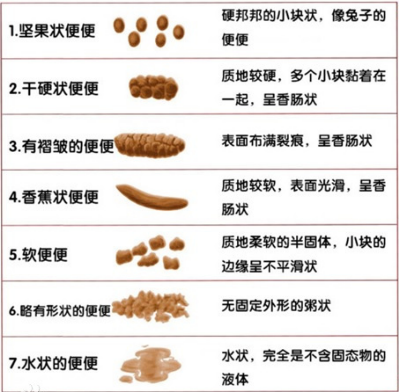Stool property | □ nut-shaped  □ dry and hard  □ lumpy  □ banana-shaped  □ soft  □ mushy  □ watery |
| Presence or absence of mental disorders such as ADHD and autism | □ No □ Yes _____________________ |
| Presence or absence of emotional pressure from school and family | □ No □ Yes _____________________ |
| Presence or absence of family changes, including death of family members, divorce of parents, moving house, birth of siblings, etc.; | □ No □ Yes _____________________ |
| Presence or absence of preferring fruit juice, tomato juice, oxalic acid-rich beverages (dark green tea, iced tea), caffeinated beverages, etc.; | □ No □ Yes _____________________ |

**Appendix 2.** Follow-up table, urination diary, defecation diary

| Date | Urinary Frequency (Morning) | Urinary Frequency (Afternoon) | Urinary Frequency (Night) | Defection Frequency | Stool Property |
| --- | --- | --- | --- | --- | --- |
|  |  |  |  |  |  |
|  |  |  |  |  |  |
|  |  |  |  |  |  |
|  |  |  |  |  |  |
